# Components of joint health and wellbeing strategies: a pan-London review of all 33 local authorities

**DOI:** 10.1186/s12889-024-20579-6

**Published:** 2024-12-18

**Authors:** Marie Line El Asmar, Manisha Karki, Bathsheba Mall, Eva Riboli-Sasco, Austen El-Osta

**Affiliations:** 1 Self-Care Academic Research Unit (SCARU), Imperial College London School of Public Health, London, UK; 2 London Borough of Hammersmith and Fulham, London, UK

**Keywords:** Health and Wellbeing, Strategy, Local authority, Public health, Policy

## Abstract

**Background:**

In the UK, Health and Wellbeing Strategies (JHWS) were introduced to identify and help address the health needs of local communities. JHWS translate a borough’s Joint Strategic Needs assessment into actionable outcomes and prioritises areas of focus, but the content and approach of JHWS may vary across different local authorities (LAs) due to demographic and priority differences.

**Objective:**

Characterise the key health and wellbeing components of existing and emerging health and wellbeing strategies of 33 local authorities in London.

**Design:**

Scoping review study with content assessment.

**Methods:**

An online search was conducted to identify JHWS documents. This search was supplemented with outreach to health and wellbeing boards to ensure that the latest documents were retrieved. A set of 62 health and wellbeing themes and words to be captured spanning 13 categories was developed by a team of researchers, and data collection was independently carried out by two researchers. Occurrences of words in the strategy documents were recorded, and a configuration matrix was created and used to determine data trends across the local authorities. A broad search of the context in which the search terms appeared was also performed to understand the trends.

**Results:**

Thirty-three JHWS were analysed to identify key search term frequencies. The analysis encompassed strategies from 2015 to 2030, ranging from 7 to 94 pages. Mental health and youth terms emerged as the two top priorities across all strategies. Other frequent terms were related to access to healthcare, inequality, employment, smoking, housing, carers, and elderly individuals. Conversely, terms related to dental health, pollution, nutrition, sexual health, green spaces, breastfeeding, and climate were less cited.

**Conclusion:**

This analysis provides insights into regional health priorities and a comprehensive overview of health and wellbeing strategies across London's local authorities. Despite certain limitations, the findings offer valuable insights for local authorities' strategic planning and future research.

## Introduction

The importance of population health and wellbeing has risen to the forefront, especially following the advent of the COVID-19 pandemic, which highlighted and exacerbated existing inequalities and disparities [1]. The World Health Organisation (WHO) states that "wellbeing exists in two dimensions: subjective and objective. It comprises an individual's experience of their life as well as a comparison of life circumstances with social norms and values" [2]. Objective life circumstances include aspects such as education, health, and social and natural environments that shape subjective experiences of one’s overall sense of wellbeing and health [2]. Subjective experiences include aspects such as positive emotions, perceived life satisfaction and meaningfulness [3]. Both physical and mental health can influence wellbeing and the relationship between health and wellbeing in a reciprocal two-way relationship [4]. There are several correlations between wellbeing and physical health outcomes, including improved immune system response, higher pain tolerance, increased longevity, cardiovascular health, slower disease progression and reproductive health [5, 6].

Self-care plays a crucial role in empowering individuals to take responsibility for their health and its integration into public health strategies is increasingly recognised as a way to promote health equity and reduce the burden on healthcare systems. Self-care, as defined by the International Self-Care Foundation, refers to the actions individuals take to maintain their health, prevent disease and cope with illness and disability. This concept is grounded in the Seven Pillars of Self-Care framework [7–9] that categorises self-care activities as related to (i) health literacy, (ii) mental wellbeing, (iii) physical activity, (iv) healthy eating, (v) risk avoidance, (vi) good hygiene practices, and (vii) the rational use of products and services.. Recent literature highlights the importance of embedding self-care into health policies to promote a more proactive approach to health management [7, 8].

In April 2008, the UK Department of Health and Care introduced the Joint Strategic Needs Assessment (JSNA) to strengthen collaboration between the National Health Service (NHS) and local government in decision-making [10]. JSNAs are used by local authorities (LAs) and borough-based partnerships to assess the needs of the local community in terms of health, care and wellbeing [10]. Health and Wellbeing Boards, which were inaugurated in 2013, were designated to create JSNAs and develop Health and Wellbeing Strategies (JHWS) to guide implementation of interventions to help reduce inequalities and improve community health by bringing together bodies from the NHS, public health and local government [10, 11]. In England, through joint health and wellbeing strategies, local authorites, Integrated Care Systems (ICSs) which span a number of LAs, and statutory bodies including NHS England are able to assess the needs of their local population and determine how these needs should be addressed [11, 12].

Population health and therefore community wellbeing depend on a wide range of determinants, some of which are beyond the reach of healthcare and local services [13]. Health and Wellbeing Boards thereby provide public health professionals with the opportunity to influence and shape public health services and activities that aim to improve community health and wellbeing.

In response to the JSNAs, local authorities use national guidance [14] to develop JHWS, which set out strategic priorities and actionable plans to address the identified needs and reduce health inequalities. The JHWS of a local authority typically covers a three to five-year timeframe and includes specific objectives for both the general population and targeted sub-populations [15]. Although national policy has a significant influence on population health, most of the prioritising and achieving of health goals are done at a local level, in collaboration with local structures and ICSs [13]. It is notable that JHWSs were introduced in 2015 a year prior to the establishment of ICSs in England.

To the authors’ knowledge, no previous attempts have been made to characterise JHWSs across the UK’s capital. There are a total of 33 local authorities in London, comprising 32 London boroughs and the City of London, which is a ceremonial county also known colloquially as the Square Mile. The primary aim of this study was to characterise the components of JHWS across London by identifying health, wellbeing and self-care themes in these statutory documents across London’s 33 local authorities.

## Methods

The document analysis followed the READ (Ready materials, Extract data, Analyse data, Distil) approach for health policy research by Dalglish et al. [16].

### Search strategy

An online search was conducted by two researchers to identify JHWS for each of the 33 boroughs in London. This resulted in the identification of an initial list of JHWS. Data collection for this study started on the 23 February 2022. After acquiring the initial list of JHWS, each LA’s health and wellbeing board was subsequently contacted via email on the 5 April 2022, requesting to share the most recent or formative JHWS. A follow-up email was sent one month later. A two-month period was allocated for responses to ensure the inclusion of the most current information available on JHWS, and data collection ended on the 19 July 2022. On the occasion that no email response was received from the board, the latest JHWS published online was used.

### Content assessment

A five-member team of researchers decided on the health and wellbeing themes to be captured, which included the following 13 categories: mental health, physical wellbeing, health services, healthy food, risk avoidance, obstetrics, living conditions, health conditions, environment, employment and education, age groups, inequality, and miscellaneous (carers, digital care, and self-care/self-management). We employed an inductive approach to allow for the emergence of new themes and categories and remained open to identifying new terms and themes that might emerge from the data. This iterative process involved pilot testing with a subset of 10 documents, during which we refined and expanded our list of search terms and categories. This refinement continued until we achieved saturation such that no new significant terms or categories were identified in the subsequent documents.

We identified items in JHWS where clear categories for action were set out in guidance on the public health responsibilities of local government. We also identified items in JHWS where individual action was needed to improve specific health outcomes. In the latter, the analysis was broadly guided by the seven pillars of the self-care framework [7–9] that categorises self-care activities as related to (i) health literacy, (ii) mental wellbeing, (iii) physical activity, (iv) healthy eating, (v) risk avoidance, (vi) good hygiene practices, and (vii) the rational use of products and services.

A specified set of potential search terms and their synonyms were developed that could be used to represent each of the broad categories that could be represented in each JHWS document. An extraction sheet, comprising a comprehensive list of search terms grouped into broader categories, was refined through repetitive revisions following pilot testing with a subset of 10 documents. This process continued until saturation of concepts and search terms was achieved. The list of 13 categories and 62 search terms used are presented in Table 1.

**Table 1:** Categories and the corresponding search terms used in document analysis

Categories	Search terms
Mental Health	Mental; Depression; Anxiety; Lonel(iness); Suicide, Self-harm; Isola(te/tion/isolated); Stress
Physical wellbeing	Physical activit(y, ies); Physical Health, Physical wellbeing; Cycl(e/ing); Walk(ing); Sport, Exercis(e/ing)
Health services	Screening; Cancer; Health check; Vaccin(e, ation), Immunisation; Admission; Dental/oral (health); Sexual health
Healthy food	Obesity; Healthy food; Nutrition; Diet
Risk avoidance	Drug/Substance; Alcohol/Drinking; Smok(er, ing)/Tobacco/Cigaratte; Violence (prevention); Crime (prevention); Domestic (violence, abuse)
Obstetrics	Pregnan(t/cy); Breastfe(ed, ing)
Living conditions	Homeless(ness); Housing; Rough sleep (ing/ers)
Health conditions	Diabet(es, ic); Dementia; Illness; Cardiovascular, heart (conditions); Long term condition
Environment	Air (e.g. quality, clean); (clean) Water; Climate (e.g. change, emergency); pollut(ed, ion)
Employment and Education	Employment; Education; Workplace
Age groups	Elderly/older (people); Adult(s); Adolescent; Young/youth/Child(ren/hood)
Inequality	Inequalit(y/ies); Access;Equit(y, able); Depri(vation, ved); Disadvantaged, vulnerable; Ethnic (Minority)/BAME
Miscellaneous	Green spaces; Digital; Self-care, Self-manag(ement), Self-help, Self-assessment, Self-reported wellbeing

### Data extraction

Data collection was carried out independently by two researchers and extracted on a data extraction form created using Microsoft Excel and piloted on a subset of JHWS strategies. The extraction template comprised five sheets (one for each ICS) containing columns for each LA within the respective ICS. These sheets included a total of 62 rows representing the 13 broader categories, with input fields for word counts/frequencies. From each JHWS, the following data were extracted: designated time frame of the strategy, number of pages, listed priorities, and search term frequencies.

Using the extraction sheet, two researchers independently searched JHWS for occurrences of each search term. Words were initially searched automatically using the computer's ctrl + F function, followed by a manual review of the documents to eliminate words that might have been inaccurately identified during the search. A standardised approach was adopted to include derivational suffixes. For instance, 'isola' was searched to encompass 'isolate, isolated, and isolation' (Isola(te/tion/isolated) Table 1. Additionally, synonyms were searched for, and frequencies were added when appropriate, such as 'physical health' and 'physical wellbeing'.

### Data analysis

**Fig. 1:**
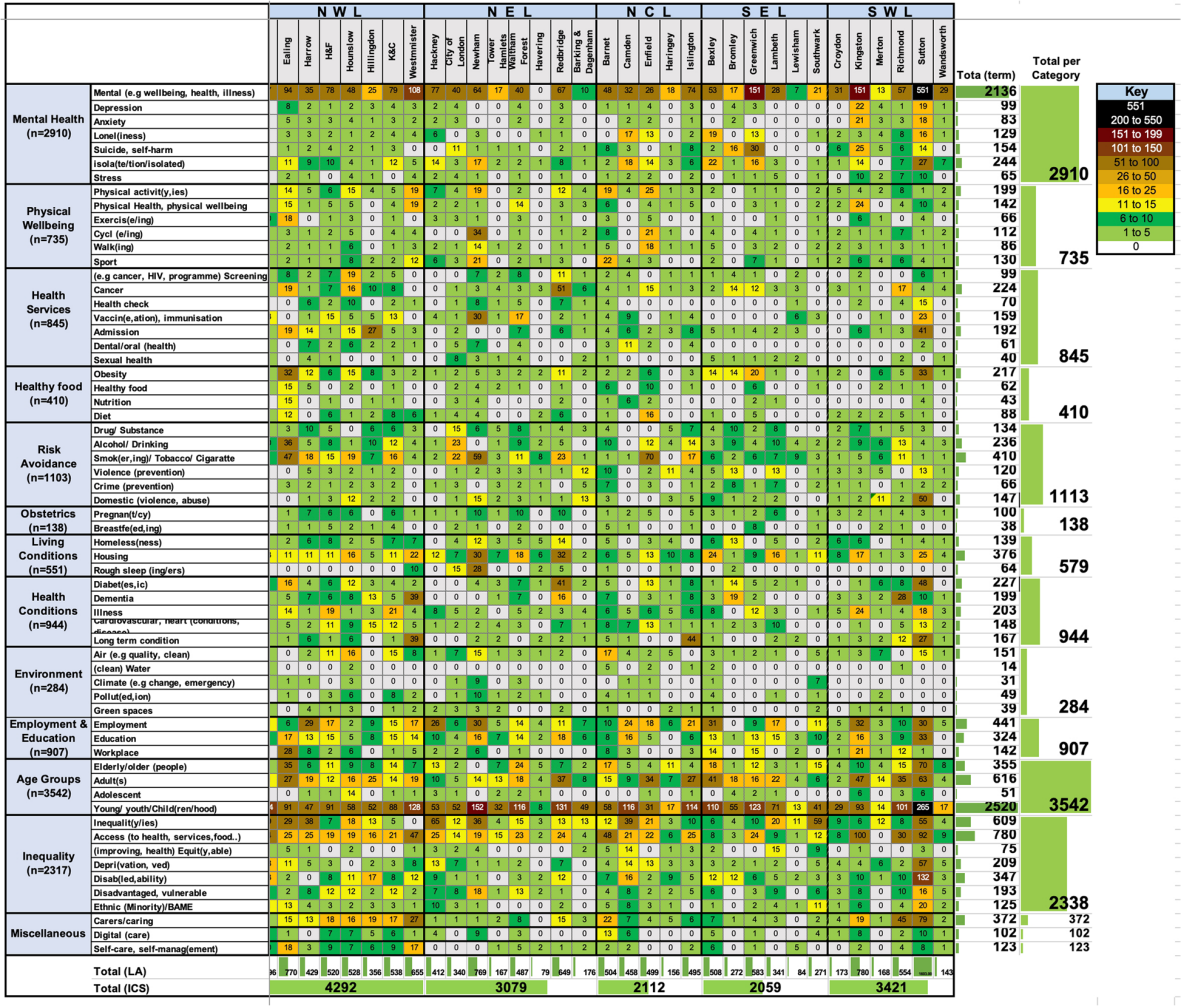
Configuration matrix and heatmap of the frequency of appearance of each search term within each local authority in London

Following data extraction, MA, MK and BM met to review the data and check for the accuracy and consistency of the methodology used. The extracted data were used to construct a configuration matrix, depicting the frequency of each search term and identifying trends in a heatmap representation (Fig. 1).

Subsequently, a broad review of the JHWS documents was conducted to understand the general context behind search terms and the quantitative data presented in the configuration matrix. Our analysis involved examining the placement and context of terms within JHWS documents. Specifically, we looked at whether terms appeared in key sections, such as executive summaries, strategic priorities or action plans, which are indicative of their strategic importance.

Additionally, we considered the narrative context to determine if a term was central to the discussion or merely mentioned in passing. This approach ensured that even terms with a single mention could be recognized for their significance if they appeared in critical sections of the documents. For example, a single mention of "climate change" within a JHWS might be found in a dedicated section outlining long-term strategic environmental health goals, indicating its importance despite its low frequency. We also conducted a broad review of the entire JHWS documents to triangulate our findings. This included using visual tools like heatmaps and configuration matrices to identify trends and patterns across different local authorities, helping to highlight both frequent and less frequent but significant terms. We aligned term occurrences with the stated priorities and action plans of each local authority to ensure a comprehensive understanding of thematic emphasis. This synthesis of insights allowed us to derive meaningful conclusions about the priorities and themes of each JHWS.

## Results

A total of 33 JHWS were analysed (one for each LA). Only 11/33 (30.3%) of LAs in London responded to our request for more information and included the latest version of JHWS. For non-responders (*n* = 23; 69.6%), we used the latest publicly available JHWS version published online. The 33 JHWS used in this analysis were dated from 2015 to 2030 and ranged in length from 7 to 94 pages, with an average of 33 pages. A breakdown of the key characteristics of each JHWS document and their listed priorities is shown in Table 2.

**Table 2:** Salient characteristics of the health and wellbeing strategy of each London borough

ICS	Local Authority	Years	No. pages	No. Priorities	Priorities
**NWL**	**Brent **[17]	2022–2027	37	6	Healthy Lives; Healthy Places; Staying Healthy; Healthy Ways of Working & Healthy Systems
	**Ealing **[18]	2016–2021	51	4	Partnership working to help improve health & wellbeing across the borough; Make every contact count with residents & in key settings such as schools & workplaces; Create & sustain an urban environment that helps people to make healthy choices; Support residents & communities to manage their health, prevent ill health & build resilience
	**Harrow **[19]	2020–2025	20	4	Start well; Live well; Work well; Age well
	**Hammersmith & Fulham **[20]	2016–2021	32	4	Enabling good mental health for all; Supporting children, young people & families to have the best possible start in life; Addressing the rising tide of long-term conditions; Delivering a high quality & sustainable health & social care system
	**Hounslow **[21]	2018–2022	32	3	Start well; Live well; Age well
	**Hillingdon **[22]	2022–2025	24	6	Support for children, young people & their families to have the best start & to live healthier lives; Tackling unfair & unavoidable inequalities in health & in access to & experiences of services; Helping people to prevent the onset of long-term health conditions such as dementia & heart disease; Supporting people to live well, independently & for longer in old age & through their end of life; Improving mental health services through prevention & self-management; Improving the way we work within & across organisations to offer better health & social care
	**Kensington & Chelsea **[23]	2016–2021	28	4	Enabling good mental health for all; Supporting children, young people & families to have the best possible start in life; Addressing the rising tide of long-term conditions; Delivering a sustainable health & social care system
	**Westminster **[24]	2017–2022	15	4	Improving outcomes for children & young people 9 our commitments; Reducing the risk factors for, & improving the management of long-term conditions such as dementia; Improving mental health outcomes through prevention & self-management; Creating & leading a sustainable & effective local health & care system
**NEL**	**Hackney **[25]	2022–2026	39	3	Improving mental health; Increasing social connection; Supporting greater financial security
	**City of London **[26]	2017–2021	21	5	Good mental health for all; A healthy urban environment; Effective health & social care integration; All children have the best start in life; Promoting healthy behaviours
	**Newham **[27]	2020–2023	80	12	Enabling the best start through pregnancy & early years; Supporting our young people to be healthy & ready for adult life; Supporting people around the determinants of their health; Developing high-quality inclusive services, ensuring equity & reducing variation; Meeting the needs of those most vulnerable to the worst health outcomes; Create a healthier food environment; Supporting active travel & improved air quality; Supporting an active borough; Supporting a Newham of communities where people are better connected & supported; Working towards a smoke-free Newham; Building a borough of health-promoting housing; Building an inclusive economy & tackling poverty
	**Tower Hamlets **[28]	2021–2025	17	6 + 5	**Six system-wide improvement principles (**Better targeting; Stronger networks; Equalities & anti-racism in all we do; Better communications; Communities first in all we do; Making the best use of what we have) + **Five ambitions for a 'Healthy Borough' (**Everyone can access safe, social spaces near their home to live healthy lives as a community; Children & families are healthy happy & confident; Young adults have the opportunities, connections, & local support to live healthy lives; Middle-aged people are supported to live healthy lives & get support early if they need to it; 5. Anyone needing help knows where to get it & is supported to find the right help)
	**Waltham Forest **[29]	2016–2020	32	6	Working with the community; Integrating health & social care; Prevention & early intervention; Reducing inequalities & tackling the wider determinants of health; Accountability & scrutiny; Parity of esteem between mental & physical health, & an increased focus on mental wellbeing
	**Havering **[30]	2019–2023	7	4	Wider determinants of health (Assisting people with health problems (back) into work; Further developing the Council/NHS Trusts as 'anchor institutions; Provide strategic leadership for collective efforts to prevent homelessness & the harm caused) + The communities we live in (Realising the benefits of regeneration for health & social care services; Improve support to residents whose life experiences drive frequent calls on health & social care service) + Lifestyles & behaviours (Obesity, Reducing tobacco harm, Early years providers, and schools/colleges as health improvement settings) + Health & social care services (Development of integrated health & social care services for CYP & adults at locality level)
	**Redbridge **[31]	2017–2021	34	3	Achieving the best start in life; Diabetes prevention & management; Mental wellbeing; Cancer survival; Living well in a decent home you can afford to live in; End of life care
	**Barking & Dagenham **[32]	2019–2023	15	3	Best Start in Life; Early Diagnosis & Intervention; Building individual & community strength
**NCL**	**Barnet **[33]	2021–2025	38	3	Creating a healthier place & resilient community; Starting, living & ageing well; Ensuring delivery of coordinated holistic care
	**Camden **[34]	2022–2030	41	3	Start well; Live well; Age well
	**Enfield**	2020–2023	41	4	Having a healthy diet; Being active; Being smoke-free; Being socially connected
	**Haringey **[35]	2020–2024	24	5	Healthy place; Start well; Live well; Age well; Violence prevention
	**Islington** [36]	2017–2020	24	3	Ensuring every child has the best start in life; Preventing & managing long-term conditions to enhance both length & quality of life & reduce health inequalities; Improving mental health & wellbeing
**SEL**	**Bexley **[37]	2020–2025	36	6	Giving children & young people the best start in life; Improving outcomes for adults & older people; Embedding prevention in all policies & practice; Creating healthy communities, workplaces, & homes; Creating healthy environments & built, green & blue spaces; creating economic independence & a thriving local economy
	**Bromley **[38]	2019–2023	36	10	Cancer; Obesity; Diabetes; Dementia; Adults mental health; Homelessness; Adults with a learning disability; Drugs & alcohol in young people; Youth violence; Adolescent mental health
	**Greenwich **[39]	2019–2024	47	4	Improving Mental Health & Wellbeing in the Royal Borough of Greenwich; Improving Healthy Weight; Live Well Greenwich – embedding a prevention approach; Health & Social Care System Development
	**Lambeth **[40]	2013–2023	42	4	Health & wellbeing is improving for all, & improving fastest for those communities with the poorest health & wellbeing; People are able to reach their full potential & to feel good about themselves; Everyone is able to make a contribution & to feel valued; People are safe from harm
	**Lewisham **[41]	2015–2018	10	3	Accelerate the integration of adult, children's & young people's care; Shift the focus of action & resources to preventing ill health & promoting independence; Support communities & families to become healthier & more resilient
	**Southwark **[42]	2022–2027	31	6	Ensure the best start in life for every child; Enable all children, young people, & adults to maximise their capabilities & have control over their lives; Create fair employment & good work for all; Ensure a healthy standard of living for all; Create & develop healthy & sustainable places & communities; Strengthen the role & impact of ill health prevention; Deliver high-quality, joined-up, person-centred health & social care
**SWL**	**Croydon** [43]	2018–2023	7	7	A better start in life; Strong, engaged, inclusive & well-connected communities; Housing & Environment to promote health; Mental wellbeing & good mental health are seen as a driver of health; A Strong Local Economy with quality, local jobs; Get more people more active, more often; A stronger focus on Prevention; The right people, in the right place, at the right time
	**Kingston **[44]	2019–2021	57	3	Provide services that make a difference; Work effectively in partnership; Influence for lasting change
	**Merton **[45]	2019–2024	24	3	Start well; Live well; Age well
	**Richmond** [46]	2016–2021	32	3	Start well; Live well; Age well
	**Sutton **[47]	2019–2024	94	3	Start well; Live well; Age well
	**Wandsworth **[48]	2015–2020	20	3	Healthy places; Targeted interventions; Mental health

The configuration matrix in Fig. 1 is a heatmap showing the frequency of occurrence of each search term per JHWS. The frequency of occurrence for the search terms used Table 1 ranged from 0 to 551, with the majority falling between 1 and 10 Fig. 1. The terms that appeared most frequently in all of London's JHWS were concerned with mental health, i.e., 'Mental' (e.g., wellbeing, health, illness)' and 'Young/youth/Child(ren/hood)' Fig. 1.

These two terms were related to the top two priorities identified (mental health and children and young people) and were among the top 50% percentile of search counts in nearly all LAs (excluding Havering); Fig. 1. The remaining search terms that appeared in the top 10 were 'access', 'inequalit(y/ies)', 'employment','smok(er, ing)/tobacco/cigarette', 'housing', 'carers/caring', and 'elderly/older' (people) Fig. 1.

In descending order, the following terms were among the 10 least cited: 'dental/oral' (health), 'pollut(ed, ion)', 'nutrition', 'sexual health', 'green spaces', 'breastfe(ed, ing)', 'climate', and (clean) 'water'. Below is an in-depth view of the document analysis with a narrative synthesis structured in categories as they appear in the configuration matrix presented in Fig. 1.

### Mental health

Excluding Havering, the second greatest emphasis across the other 32 LAs was concerned with mental health. The search term "mental" (e.g., wellbeing, health, illness) was the second most frequently encountered term across all JHWS (*n* = 2136), following Young/Youth/Child(ren/hood) (*n* = 2520). Sutton, Kingston, Greenwich and Westminster had the most frequent mentions of mental health terms across London. In Havering's strategy, there was only one mention of 'loneliness' and 'isolation,' with no reference to general mental wellbeing terms. Compared to other ICSs, depression and anxiety were more frequently mentioned in SWL, especially in Kingston and Sutton JHWS. Overall, 'isolation', 'loneliness', and 'self-harm/suicide' appeared to be mentioned more frequently than the other specific terms that were searched (depression, anxiety, loneliness, suicide/self-harm, stress). Notably, suicide and self-harm were mentioned the most by Greenwich, followed by Kingston and Bexley. Ten local authorities did not report at least three out of six more specific terms related to mental health Fig. 1. Generally, the context of the term 'mental health' was linked to building behaviours that reduce the number of people who develop mental health disorders in the long term (i.e., self-care/prevention), securing improved access to mental health services, and sustaining good mental health for all.

### Physical wellbeing

'Physical activity' was the 20^th^ most frequently occurring term (*n* = 199). Overall, the term 'physical*' was more prevalent in the JHWS of seven LAs (Ealing, Hounslow, Westminster, Newham, Redbridge, Barnet and Enfield)**,** whereas two LAs (Bromley and Lewisham) did not mention physical activity or other related search terms in their strategies**.** Most LAs aimed to reduce factors for long-term health conditions by encouraging physical activity in all age groups and decreasing the impact of the pandemic (where relevant) on physical health, including the impact on children and young people (C&YP) health and wellbeing. Other key objectives included ensuring safe outdoor spaces for exercise purposes and enhancing walkable and cyclable routes.

### Health services

'Sexual health' and 'dental/oral health' were among the least frequently encountered terms in JHWS pan London, ranking 6^th^ and 10^th^ least common, respectively. There was no mention of 'sexual health' and 'dental/oral (health)' in 16 (48%) and 17 (51%) JHWS, respectively. “Dental or oral” were frequently mentioned in North London LAs, but excluding Sutton were otherwise absent in almost all South London ICS strategies. There was no mention of 'sexual health' in NCL and SWL (excluding Barnet, Richmond, and Wadsworth). Where dental health was mentioned, strategies included improving children's oral health, establishing good oral hygiene habits in early years, setting up dental health buses, and developing an integrated health promotion offer for children to improve dental health. Strategies for sexual health included ensuring timely access to sexual health services, reducing the number of people diagnosed late with human immunodeficiency virus (HIV), detecting and diagnosing sexually transmitted illnesses effectively, and supporting healthy sexual behaviours.

Excluding Sutton, compared to other ICSs, NWL JHWS used the term 'admission' more frequently in the context of reducing overall emergency admissions due to alcohol, falls, dementia and long-term condition-related admissions and reducing avoidable admissions following discharge from hospitals. Apart from Newham, the Waltham Forest and Sutton, which referenced vaccination most frequently, mentions of 'vaccination' or 'immunisations' were more frequent in NWL JHWSs’ compared to other ICSs. Among the aims were to establish vaccination sites in areas where vaccine hesitancy was greatest and to improve the uptake rates of COVID-19, influenza, human papillomavirus (HPV) and childhood vaccinations. Overall, 'cancer' ranked as the 16th most frequently occurring term, with a notably higher emphasis in Redbridge's strategy. Ten LAs did not use the term 'screening', which mostly referred to increasing uptake and awareness of bowel, breast and cervical cancer screening, screening advice on sexually transmitted infections and conception, and preventing chronic diseases through early screening.

### Healthy food

Overall, 'obesity' was the 17^th^ most commonly used term (*n* = 217), with Ealing and Sutton citing the term most frequently. Compared to other LAs, Ealing had the greatest emphasis on healthy eating, diet and nutrition overall. Three LAs (Haringey, Lewisham, and Kingston) did not mention 'obesity'. When healthy food or obesity was mentioned, it was mostly in the context of reducing childhood obesity, delivering weight management action plans for all ages, and providing targeted support for those most at risk. A range of other strategies were proposed, including increasing access to healthy food options in high streets, near schools and in deprived areas and encouraging people to grow their own food. There was also mention of reducing premature deaths by improving diets, ensuring food security, and developing integrated health promotion programs for children and families.

### Risk avoidance

Of the searched terms in this category (drugs/substances, alcohol/drinking, violence, crime, domestic violence), smoking received relatively higher mentions, particularly in Ealing, Enfield and Newham. Moreover, 'smoking' ranked as the seventh most frequently encountered term across all JHWS. Haringey's JHWS was the only strategy that did not mention terms related to smoking. Smoking cessation strategies largely focused on improving access to services, reducing the prevalence of smoking overall and in certain groups (such as pregnant women and manual workers), reducing smoking in families, and reducing the uptake of smoking among children and youth. Despite alcohol being the 14^th^ most occurring term overall and the 2^nd^ most occurring term in this category, it was missing from three JHWS (Newham, Barking and Dagenham, and Camden). Drugs/substances were not mentioned in seven JHWS. Interventions to address alcohol abuse included using licensing powers to regulate alcohol and focusing on areas of deprivation where alcohol use is higher. Compared to all other strategies, Sutton had the highest number of references to tackling/reducing domestic abuse (*n* = 50), followed by Merton, Barking and Dagenham, Newham, and Hounslow. Generally, there was a higher mention of violence/crime prevention in some LAs of SEL and NCL (namely, Barnet, Haringey, Bromley, Sutton and Lambeth), which included tackling knife crimes and youth violence.

### Obstetrics

Overall, JHWSs had a lower occurrence of the terms 'pregnancy' and 'breastfeeding', with breastfeeding ranking fourth in frequency overall. Five strategies (Brent, Havering, Haringey, Barking and Dagenham, and Lewisham) did not mention either term. Breastfeeding was promoted through counselling, community promotion, breastfeeding cafes, and baby-feeding workshops. Among the other topics discussed were support for teenage pregnancies, early access to maternity services, reducing pregnancy terminations, identifying depression during and after pregnancy, and promoting wellbeing. A particular focus was reducing smoking prevalence in pregnant women across a majority of JHWS.

### Living conditions

"Housing" ranked as the 8^th^ most frequently occurring term overall, appearing in all 33 JHWS, and notably emphasised in Newham and Redbridge. Bromley and Newham appeared to have the highest mentions of homelessness and rough sleeping, while only seven JHWS did not refer to homelessness. Strategies to promote living conditions included improving housing standards, providing affordable housing, ensuring that green spaces and water features were integrated into new developments, and promoting wellbeing on social housing estates. Other strategies aimed to address homelessness and assist landlords in connecting residents with financial help, providing support for residents in care homes and sheltered housing, and developing integrated housing and social care services.

### Health conditions

When compared to other ICSs that had a higher number of strategies with missing terms, both NWL, NCL and SWL ICSs tended to include almost all the terms searched under this specific category. Diabetes (along with cancer and obesity) was among the top 20 most frequently occurring terms overall. Strategies aimed at improving the management of long-term conditions, particularly diabetes, dementia and cardiovascular disease (CVD), included a focus on prevention programmes, targeted support and early identification through health checks to be offered in a range of settings. Noticeably, some LAs in NEL and SEL did not mention the term 'dementia'. Strategies to tackle dementia involved integrated service approaches, encouraging awareness, improving the quality of dementia-related care, reducing dementia-related hospital admissions, and promoting dementia-friendly communities.

### Environment

'Clean water' and 'climate' were the two least occurring terms overall, whereas 'pollution' and 'air quality' were more frequently found. Less than one-third (*n* = 7; 21%) of LAs mentioned clean water. In terms of air quality, most LAs focused on improving it, decreasing the impact of poor air quality on heart and lung patients, establishing a sustainable system to improve air quality, developing low-emission, low-traffic neighbourhoods, improving public transportation, installing more electric charging points, and launching anti-idling campaigns. Green spaces were mentioned less frequently in strategies from South London, with a relatively higher occurrence in strategies from North London, where the aims were to enhance green and open spaces that encouraged physical exercise and improve mental health.

### Employment and education

'Employment' and 'education' were the 6^th^ and 12^th^ top terms overall, respectively. Although most LAs reference education and employment, mention of the workplace is relatively lower. Several objectives were set, such as promoting fair wages, youth employment in creative industries, creating employment opportunities for young people, and providing employment support for isolated groups and for residents with mental health conditions and learning disabilities. Various educational objectives were identified, including promoting child development through parent education, assisting children with disabilities in accessing education, promoting wellbeing in education and workplaces, educating patients, and supporting the development of mental health and physical health education programs. Furthermore, strategies included creating and maintaining healthy workplaces, introducing voluntary weight watching, exercise, and smoking cessation programs at work, developing pathways to mental health support from employment and promoting suicide prevention training in the workplace.

### Age groups

Youth/children-related search terms ranked as the most frequent across all strategies, indicating a great emphasis on children and young people (C&YP) in all strategies. Among many other broad objectives, strategies included maintaining the physical and mental wellbeing of C&YPs, ensuring a good start in life for all children, tackling youth violence, increasing support for C&YPs exposed to domestic violence, enhancing school readiness and oral health, addressing poverty, protecting vulnerable children, and promoting opportunities through digital platforms. Almost all strategies had objectives relating to elderly individuals, such as improving their mental health and wellbeing, providing early support through effective signposting to services, supporting them in ageing well, supporting them against isolation through social programmes, helping them remain healthy and independent, and addressing food security and financial hardship.

### Inequality

Except for one JHWS (Westminster), all referenced the term inequality. Nearly all JHWS extensively covered topics such as access to healthcare, nutritious food and a diverse array of services. Sutton had a significantly higher frequency of mentioning disability compared to other LAs (*n* = 132). Ethnic minorities were mentioned in all NWL JHWS with the intention of addressing physical and mental health disparities among ethnic groups, decreasing certain health conditions that are more pervasive, ensuring a good start in life irrespective of background, and reducing unplanned hospitalisations in ethnic communities. Half of the local authorities in NEL did not mention ethnic minorities in their JHWS. There were several objectives related to inequalities, including addressing social inequality and determinants of health, maximising opportunities for special needs children, improving services for vulnerable groups, enhancing access to primary care and screening tests, providing housing for the homeless, creating jobs and environments with low crime rates, providing treatment for early-stage diseases, and providing mental health referrals.

### Miscellaneous

'Carers' was a top term appearing in most strategies, excluding two LAs (Havering and Lewisham). The context around carers included using digital platforms to support carers, address health inequalities, identify carers early, provide clear, consistent, and accessible information about the support available, and assist caregivers in maintaining and improving their health, wellbeing, and social inclusion. Terms related to self-management, self-care and digital inclusion appeared in the bottom 50^th^ percentile of the list of overall frequencies. NWL strategies offered context for self-care and digital inclusion more consistently compared to other ICS strategies. Self-care and self-management terms appeared in the context of developing a future system centred around self-care to prevent and delay the need for care, piloting services for managing chronic diseases such as diabetes, and promoting self-care through primary care, social care, and mental health services.

Regarding digital support and inclusion, among the objectives was to promote digital services that help young people with mental health conditions who would not otherwise seek therapy face-to-face. Initiatives such as digital community hubs, which offer devices and the training necessary to utilise them, were set up to close the skills, connectivity, and accessibility gaps among residents.

## Discussion

The present study offers a comprehensive exploration of JHWS across all 33 LAs in London, providing novel insight into themes within these documents. To the authors' knowledge, this is the first study to characterise the components of JHWS across London to help identify health, wellbeing, and self-care themes.

### Summary of principal findings

The content assessment of London JHWS revealed a nuanced landscape of health and wellbeing priorities. The two most prominent themes include a strong focus on improving outcomes for the C&YP and fostering mental health wellbeing within communities. Mental health appeared to be a significant concern across most LAs, with varying emphasis among LAs. C&YP were a central focus across strategies.

Other prominent themes revolved around addressing health inequalities, access to health services, employment, smoking cessation, housing, support for carers and elderly individuals, and tackling obesity. On the other hand, certain areas are relatively less emphasised, such as dental and oral health, sexual health, pollution, nutrition, green spaces, and breastfeeding.

Physical activity was a key priority, especially linked to outdoor activities, potentially influenced by pandemic-related limitations on indoor gatherings. However, variations in physical activity levels are possibly tied to factors such as access to green spaces. With the exclusion of Bromley and Lewisham in SEL, most LAs pledged to promote physical activities, which was the 20th most frequently occurring term and was also cited as being a way of encouraging healthier lifestyles with an emphasis on outdoor activities.

There was a significant mention of obesity, cancer, and diabetes, particularly in certain boroughs, which highlights their prevalence as health concerns. Conversely, terms such as dental and oral health, sexual health, and certain aspects of cancer screening lack prominence, potentially due to local ICSs overseeing these areas. It was surprising that dental or oral health and sexual health were among the least common terms found in JHWS, and almost half of the JHWS did not mention them at all, although a quarter (26%) of 5-year-olds in London suffer from decay [49] and STIs remain an important public health problem in London [50].

### Interpretation and study implications

The findings of this study shed light on the varying priorities and approaches that LAs in London undertake to address the main public health concerns. Every LA has a responsibility to develop a health and wellbeing strategy which involves a wide-ranging assessment of council priorities and existing services and how these could be shaped to deliver on themes identified through the JSNA. The findings indicate that all the JHWS took a high-level perspective, with the local characteristics of each borough reflected in the overall direction and focus of each strategy.

"Youth"-related terms were the most prevalent throughout all strategies, which becomes more significant in light of the worldwide focus on recognising the value of investing in the health and wellbeing of future generations [51–53]. This investment is seen as pivotal for establishing strong, healthy foundations for the youth and fostering a more sustainable and equitable world [51]. The prevalence of mental health as a second most prominent focus also highlights the growing recognition of its significance. In 2014, 1 in 6 individuals aged 16 + in England experienced poor mental health, with rates rising after the pandemic [54]. Similarly, rising prevalence is seen in C&YP individuals [55, 56]. The NHS Long Term Plan aims to elevate mental health care to the status of physical health services, and although mental health spending is not protected, Clinical Commissioning Groups are required to meet the’Mental Health Investment Standard’ [57]. "The Five Year Forward View for Mental Health" by NHS England focuses on prioritising improved service access to address not only the treatment but also the prevention of mental health disorders [58].

Another interesting observation is the prevalence of physical activity as a key priority, especially linked to outdoor activities, possibly influenced by pandemic-related limitations on indoor gatherings. However, variations exist, potentially linked to factors such as access to green spaces, among others. In strategies that were published post-pandemic, this could be linked to the limitations placed on congregating inside enclosed, private spaces. An alternative interpretation could be that more emphasis was placed on the type of activity undertaken, such as walking or cycling, which may be more prevalent in Bromley, an outer London borough with significantly more access to green space central locations.

The findings highlighted disparities in the attention given to different aspects of healthcare, such as sexual and dental health, indicating potential areas for more comprehensive strategies. This could be due to differing unmet needs between populations that strategies aim to meet. The extensive coverage of health conditions, including diabetes, cancer and obesity also highlights the continuous need to manage and prevent these conditions which are among the most prevalent in the UK [59]. Conversely, the lower occurrence of other aspects of healthcare, such as dental and sexual health, may indicate either an oversight by ICSs or a potential gap in awareness or prioritisation. Further exploration is needed to understand the underlying factors and implications for healthcare planning and resource allocation.

Smoking received significant attention and was addressed in most JHWS, including a focus on smoking in pregnant women, implying a commitment to address this preventable public health issue, considering that the UK has one of the lowest smoking rates in Europe, with 1 in 6 adult smokers [60]. Housing and employment were also highly referenced, as poor housing conditions and unemployment are recognised as underlying causes of poor health [60]. The infrequent mention of 'clean water' and 'climate' raises questions as to whether they are perceived as less pressing concerns, ot mentioned in other local strategy documents centred around climate change and the environment. Another prominent theme was addressing inequalities and access to different services, reflecting a commitment to address this social determinant of health.

The comparative analysis of JHWS highlighted in at least two styles. One approach, categorised as "life courses", is to use a strapline style such as "Healthy Lives, Healthy Places, Staying Healthy, Healthy Ways of Working, Healthy Systems", illustrated by the example of Brent, with a clear link to maintaining health as a central tenet. In contrast, in others such as Ealing, there is a more defined style with four key priorities set out in broad detail. The first is concise, designed to be aspirational, memorable and clearly linked by the term "health", to capture in a few words an intent to promote the benefits of a healthy life. The second adopts a more thematic approach, expansively framing the strategy's aims, and although 'health' is a term featured throughout, each priority is contextualised. Notably, the absence of terms does not imply neglect of topics but might be subsumed under broader themes.

A binary analysis of the frequency of a term offers several observations. First, the absence of the term does not infer that the issue is not covered more broadly under key or emerging themes. Second, it highlights the absence of nominal terms in some JHWS, for example, "BAME" (black and Asian minority ethnic). Third, the configuration matrix has the potential to offer many more observations if we are able to interpret binary patterns in the context of overarching and emerging themes. A correlation to the frequency of occurrence is not an indication of the robustness of the strategy, nor is it an indication of the breadth of themes presented. Contextualisation allowed greater latitude for interpretation. Aspects such as the length of the document, the adopted style, format, use of graphics or the exclusion of prominent public health terms are not, in insolation, indicators of quality nor its effectiveness as an intervention.

The evidence synthesis highlighted both strengths and weaknesses in the different strategies and identified key areas that could be expanded and refined. Examining the effectiveness would be problematic, as it would not be possible to obtain metrics to indicate outcomes to form meaningful conclusions as to whether these have been successful. The study's implications, therefore, extend beyond individual strategies and highlight both common priorities and variations. This could potentially inform collaborative efforts and knowledge sharing among LAs to arrive at more inclusive and effective public health interventions.

Our findings highlighted a strong alignment between the themes identified in the JHWS and the priorities outlined in the JSNA. Both documents emphasize key areas such as mental health, physical wellbeing, health services, and addressing inequalities, reflecting the significant health challenges and community needs identified by local authorities. The JJHWS themes are tailored to specific demographic needs highlighted in the JSNAs, ensuring a responsive approach to local health issues, whereas emergent themes such as digital health and self-care in the JHWSs indicate a proactive stance, addressing the evolving public health trends and integrating innovative health promotion strategies. This comprehensive approach ensures that JHWSs are effectively based on JSNA findings while also adapting to future health needs.

It is important to recognize that while JSNAs provide a comprehensive and up-to-date assessment of the health and wellbeing needs of the local population, JHWS are strategic documents outlining the priorities and actions based on these assessments. However, these documents are not always updated simultaneously. JSNAs are typically reviewed and updated more frequently to reflect the most current data and emerging health needs, whereas JHWS often have longer timeframes and may not be revised as regularly. This discrepancy in update schedules can potentially lead to misalignments where the strategic priorities and actions outlined in the JHWS may not fully reflect the most recent findings and emerging trends identified in the latest JSNAs. Acknowledging this potential issue is crucial for understanding the dynamic nature of health and wellbeing planning and the need for continuous review and adjustment of strategies. To mitigate this, local authorities should strive for periodic reviews of their JHWS to ensure they remain aligned with the most current JSNA data. This adaptive approach can help maintain the relevance and effectiveness of health and wellbeing strategies, ensuring they address both persistent and emerging health challenges in the community.

Notably, both the JSNA & JHWS documents are largely shaped by central guidance issued by the Department of Health and Social Care. However, while this guidance provides a structured framework for health and wellbeing planning, it is important to consider whether it is sufficiently flexible to address the varied needs of local populations. In particular, the level of prescriptiveness in the guidance may limit the ability of local authorities to tailor their strategies to emerging public health trends. Future revisions of this guidance could benefit from a more adaptable approach that balances national priorities with local flexibility.

### Emphasis on personal empowerment, choice, prevention and self-care

Self-care as a concept is rapidly gaining recognition as it is closely linked to personal empowerment, choice and prevention, and is principally concerned with emphasising the active participation of individuals in maintaining and improving their own health and wellbeing. This study prompts a critical examination of self-care and the extent to which each of the seven pillars of self-care is represented in JHWS.

All seven pillars of self-care [7–9] were emphasised to varying degrees in JHWS overall [9]. The category of risk avoidance, which included binary references to alcohol and substance misuse, smoking, violence/crime (prevention) and domestic abuse, links to every pillar but involves a higher level of self-awareness or agency, as an individual's attitude towards risk-taking behaviour is a critical factor in self-care. Other categories do not directly link to any of the pillars, for example, pollution (and variations), which includes terms such as access to clean air/quality, clean water and climate change, or the category grouping employment, education and workplace. However, these socioeconomic and environmental factors constitute social determinants of health that impact the health of individuals and may influence both the levels of self-care capability of individuals as well as the effectiveness of their self-care behaviours. Following a socioecological approach, the promotion of self-care requires addressing not only individual behaviours but also interpersonal, institutional, community and policy levels to be efficient [61].

Overall, the evidence synthesis identified areas for strategic refinement and expansion to ensure greater integration of evidence-based self-care policies in JHWS. Integrating self-care principles into JHWS presents a promising avenue for enhancing public health outcomes and promoting a holistic approach to wellness. Incorporating self-care into JHWS can also empower individuals to take greater responsibility for their health, fostering a sense of ownership and agency. By promoting self-awareness and education, JHWS can encourage healthier lifestyles, early detection of health issues, and more proactive management of chronic conditions. Embedding self-care in JHWS also aligns with the shift towards preventive healthcare and can alleviate the burden on healthcare systems by reducing avoidable hospital admissions and healthcare costs. Effective integration of self-care into JHWS requires addressing several challenges, including finding ways to ensure equitable access to self-care resources, especially for marginalised and underserved populations. Tailoring self-care initiatives to diverse cultural, socioeconomic and educational backgrounds is also crucial to prevent exacerbating health inequalities, whereas fostering a culture of self-care demands a shift in public perception and behaviour, necessitating comprehensive public awareness campaigns and education initiatives.

To successfully consolidate self-care into JHWS, a multipronged approach is needed, including collaborative efforts between LAs, healthcare providers, community organisations and educational institutions, to facilitate the development and dissemination of accessible and culturally sensitive self-care resources. Harnessing the power of social networks and peer support groups can also foster a sense of community and motivation for sustained self-care behaviours, whereas digital first platforms, such as mobile applications and websites, can also serve as valuable tools for delivering information, resources and personalised self-care plans to a wider audience. Embedding self-care within existing health services can enhance its visibility and acceptance. Incorporating self-care components into routine health assessments, clinical consultations, and community health programs can encourage individuals to adopt self-care practices as an integral part of their wellbeing journey, but this will be largely dependent on the extent to which health and care systems are able to measure an individual’s self-care capability [62].

### Strengths and limitations

A key strength of this study lies in its pan-London scope, comprehensively examining all 33 LAs and their JHWS across five ICSs. The content assessment provides a valuable snapshot of key themes, aiding future strategy development while highlighting the variation in borough- and subregional-level population needs. The emergent themes highlighted may help inform future JHWS by indicating the degree to which they are underpinned by a robust assessment of needs and offer LAs the opportunity to share examples of good practice in developing their strategies.

To minimise human error, two researchers cross-checked the results of the content assessment of each JHWS; however, the approach used in the content assessment was pragmatic and not systematic, thus limiting the areas of focus chosen in the analysis while expanding the scope. The principal limitation of this study was that we were only able to engage with a third of health and wellbeing boards across the capital, As some of the strategies were out of date or in draft form, this may have impacted the quality and relevance of our analysis, whereas some strategies that were published after 2020 offered a post-pandemic lens confounding a direct comparison with strategies published pre-pandemic.

The choice of measuring units for analysis and the potential fragmentation of content analysis might affect findings, whereas an inherent inability to search all synonyms within each category and no anonymity of JHWS sources also limited the quality of our analysis. Despite these limitations, the pragmatic content assessment provides an objective pan-London overview and sheds light on the various dimensions of health priorities and strategic planning at both the borough and subregional levels.

A future study could be framed within the context of the Mayor of London’s Health Inequalities Strategy [63], which provides a city-wide framework for addressing health disparities and promoting equitable health outcomes by considering the six themes of (i) Healthy Children, (ii) Healthy Minds, (iii) Healthy Places (iv), Healthy Communities, and (v) healthy living.

## Conclusion

This study offers a holistic view of the health and wellbeing strategies of London's 33 local authorities highlighting diverse priorities and the necessity to tailor approaches to meet specific local needs. The study's findings contribute valuable insights for strategic planning and future research endeavours. Further incorporation of health promotion and primary prevention strategies coupled to pledges for action by local governments on cross-cutting issues such as tackling air pollution and other determinants offers a transformative approach to public health, emphasising individual empowerment, preventive strategies and improved health outcomes.

## Data Availability

The data supporting this study's findings are available from the corresponding author upon reasonable request.

## Abbreviations

C&YP: Children and young people
C&YP: Children and young people
CCGs: Clinical Commissioning Groups
CVD: Cardiovascular disease
JHWS: Joint Health and Wellbeing Strategy (or strategies)
HIV: Human immunodeficiency virus
HPV: Human papilloma virus
ICSs: Integrated Care Systems
JSNAs: Joint Strategic Needs Assessments
LAs: Local Authorities
NCL: North Central London
NEL: Northeast London
NHS: National Health Service
NWL: Northwest London
READ: Ready materials, Extract data, Analyse data, Distil approach
SEL: Southeast London
SWL: Southwest London
WHO: World Health Organisation
